# Coupled Effects of Natural and Anthropogenic Controls on Seasonal and Spatial Variations of River Water Quality during Baseflow in a Coastal Watershed of Southeast China

**DOI:** 10.1371/journal.pone.0091528

**Published:** 2014-03-11

**Authors:** Jinliang Huang, Yaling Huang, Zhenyu Zhang

**Affiliations:** 1 Coastal and Ocean Management Institute, Xiamen University, Xiamen, People’s Republic of China; 2 Fujian Provincial Key Laboratory for Coastal Ecology and Environmental Studies, Xiamen University, Xiamen, People’s Republic of China; University of Sydney, Australia

## Abstract

Surface water samples of baseflow were collected from 20 headwater sub-watersheds which were classified into three types of watersheds (natural, urban and agricultural) in the flood, dry and transition seasons during three consecutive years (2010–2012) within a coastal watershed of Southeast China. Integrating spatial statistics with multivariate statistical techniques, river water quality variations and their interactions with natural and anthropogenic controls were examined to identify the causal factors and underlying mechanisms governing spatiotemporal patterns of water quality. Anthropogenic input related to industrial effluents and domestic wastewater, agricultural activities associated with the precipitation-induced surface runoff, and natural weathering process were identified as the potential important factors to drive the seasonal variations in stream water quality for the transition, flood and dry seasons, respectively. All water quality indicators except SRP had the highest mean concentrations in the dry and transition seasons. Anthropogenic activities and watershed characteristics led to the spatial variations in stream water quality in three types of watersheds. Concentrations of NH_4_^+^-N, SRP, K^+^, COD_Mn_, and Cl^−^ were generally highest in urban watersheds. NO_3_^–^N Concentration was generally highest in agricultural watersheds. Mg^2+^ concentration in natural watersheds was significantly higher than that in agricultural watersheds. Spatial autocorrelations analysis showed similar levels of water pollution between the neighboring sub-watersheds exhibited in the dry and transition seasons while non-point source pollution contributed to the significant variations in water quality between neighboring sub-watersheds. Spatial regression analysis showed anthropogenic controls played critical roles in variations of water quality in the JRW. Management implications were further discussed for water resource management. This research demonstrates that the coupled effects of natural and anthropogenic controls involved in watershed processes, contribute to the seasonal and spatial variation of headwater stream water quality in a coastal watershed with high spatial variability and intensive anthropogenic activities.

## Introduction

River water quality has become one of important concern worldwide. On the one hand, rivers constitute the main water resource for drinking, irrigation, and industrial purposes in inlands [1]–[2]. On the other hand, as receiving water bodies, rivers assimilate or carry industrial and domestic wastewater, and runoff from agricultural fields, roadways and streets, thereafter discharging them into downstream estuarine and coastal water [3]–[4]. It is reported that nearly 80% (4.8 billion) of the world’s population (for 2000) lives in areas where either incident human water security or biodiversity threats exceed 75^th^ percentile [5]. In addition, water quality all over the world presents a trend of increasingly severe deterioration [6]. As such, it is important to have reliable information on river water quality for water resource management from the local to global scale. This necessity is even more pronounced in coastal watersheds due to escalating environmental pressure and their special role in regional ecosystem services.

Water quality is affected by a combination of natural factors (e.g. precipitation, temperature, bedrock, soil, terrain) and anthropogenic factors (e.g. agricultural practices, domestic wastewater/industrial influent) [7]–[9]. Understanding how anthropogenic and natural factors control water quality and how the relationships changes over time and space will help water resource managers to target appropriate scales and factors for the improvement of their water quality management efforts.

The factors and processes involved in natural and anthropogenic controls govern the seasonal and spatial variability in stream water quality in watersheds. It is important to determine the watershed processes that regulate stream water quality under increasing pressure from natural and anthropogenic disturbance [5], [10]–[13]. Hydrological and biogeochemical processes are two important mechanisms to explain the seasonal variations of water quality in the watersheds [3], [14]–[15]. The nutrients are temporarily stored in the ground surface, vadose zone or in the groundwater and then transported into stream via subsurface water or precipitation-induced surface runoff [15]–[16]. The biogeochemical processes including in-stream immobilization, denitrification, mineralization, and bedrock weathering, associated with the hydro-meteorological regime, also determine the temporal variability of stream water quality [15], [17]–[18].

On the other hand, anthropogenic activities and watershed characteristics drive spatial variability in stream water quality in the watersheds. Watershed land use impacts water quality through nonpoint sources, which are major contributors of pollution to the catchment-coast continuum [19]–[20]. Linkage of land use pattern and water quality has been well documented for developing watershed management practices [7], [21]–[23]. In addition, the point sources of nutrients, which are usually synonymous with domestic wastewaters and industrial effluents [24]–[26], contribute greatly to stream water quality degradation in developing countries with relatively low wastewater treatment capacity [27]. The watershed characteristics including topography and bedrock geology constitutes the important natural factors that drives the spatial variation in stream water quality [21], [28]. The higher slope variability leads to higher rates of erosion, which subsequently increase the rates of particulate matter entering the watershed [29]. The chemical composition of streams is influenced by distinct bedrock type and the distribution of Ca^2+^ and Mg^2+^ concentrations in rivers depends strongly upon weathering of soil and bedrock geology [30]–[31].

The coupled effects of natural and anthropogenic controls, together with underlying hydrologic and biogeochemical processes, contribute to the seasonal and spatial variation of stream water quality. Magnitude of in-stream N immobilization is controlled by stream order and headwater streams are important sites for N processing and retention [32]–[33]. Martin et al. (2004) reported that high subsurface flow with high nitrate concentration during high water periods and active denitrification during low water period explained the higher streamwater nitrate concentration in winter than in summer in a watershed in France [15]. Duan and Kaushal (2013) found that Peak inorganic N concentrations occur throughout the winter and decline considerably during the growing season due to the effect of N immobilization in the Chesapeake Bay watershed [18]. Meantime, they pointed out that there is potential to increase winter N delivery to streams because of human activity, e.g. periodic agricultural activities. Bowes et al. (2005) found that streams receiving wastewater effluent typically show a characteristic pattern of high P concentration during summer low flow and more diluted concentration during winter storm events in an English catchment [34]. Rothwell et al. (2010) found that the elevated Mg^2+^ concentrations in the lowlands are due to underlying geology, rather than urban or arable land use [12].

The relative influences of the natural and anthropogenic factors change over the range of temporal and spatial scales investigated, and this results in a pressing challenge for studies on the mechanism determining the variability in river water quality, especially in the situation that water quality in most Chinese rivers and groundwater sources is poor and declining under pressures from industrial and municipal wastewater discharges and NPS pollution from agricultural and aquacultural runoffs of fertilizers, pesticides and manure and freshwater quality is a prime concern in China, especially in the relatively developed regions such as the eastern coastal areas of China [27], [35].

Analyzing the spatiotemporal variations in stream water quality generally relies on multivariate statistical techniques combined with GIS and remote sensing. Multivariate statistical techniques including principal component analysis (PCA) and redundancy analysis are useful for data reduction and interpretation of apportionment of the pollution sources [3], [36]. The increasing availability of remotely-sensed data enables landscape-water quality studies to be more easily performed on both local and regional scales [22], [37]. The conventional ordinary least square (OLS) method combined with GIS is the main method for estimating the empirical relationships between ambient water quality parameters and watershed characteristics [2], [38]. Recently, a powerful spatially statistical method, the geographically weighted regression technique, was developed and applied to examine the spatially varying relationships between land use and water quality [23]. [39]–[41]. However, few studies consider the spatial dependence of water quality explicitly. Moreover, many previous studies do not examine both spatial and temporal change simultaneously [42]–[43], which make it hard to fully uncover the spatiotemporal variation of river water quality.

The Jiulong River watershed (JRW), a typical medium-sized subtropical coastal watershed, has experienced continuing degradation in water quality over the last 20 years. This plays an important role in the region’s economic and ecological health. A better understanding of the spatiotemporal variations in water quality and the underlying mechanisms are critical for regional water quality management. The primary objective of the present study is to examine the coupled effects of anthropogenic and natural controls on the seasonal and spatial variations in water quality in 20 headwater watersheds of the JRW. We test two hypotheses: (1) anthropogenic activities, combined with the hydrological and biogeochemical processes, contribute to the seasonal variability in stream water quality; (2) coupled effects of anthropogenic activities and watershed characteristic, result in the spatial variability in stream water quality.

## Materials and Methods

### 2.1. Ethics Statement

No specific permits were required for the described field studies and our field studies did not involve endangered or protected species.

### 2.2. Study Area

**Figure 1:**
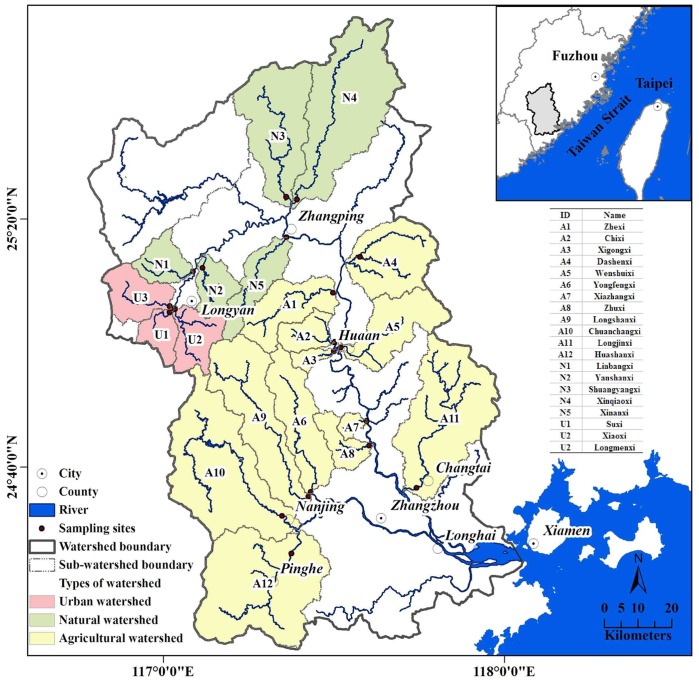
Sampling sites in the watershed studied.

The JRW covers about 14,700 km^2^ in the eastern coastal area of China (Fig. 1). The watershed includes the North and West Rivers, which meet in Zhangzhou, and produce an annual flow of twelve billion cubic meters into the Jiulong River estuary and the Xiamen-Kinmen coastal waters. It is situated in a subtropical zone with a monsoon climate: the annual average temperature is 19–21°C, and annual precipitation averages 1400–1800 mm, of which 70% occurs between April and September. Red earth and lateritic red earth are the main soil types in the JRW, with pH values ranging from 4.0 to 4.8 and a mean value of 4.5. The upstream region is mountainous and 68% of the watershed has a topographic slope in excess of 18% [44]. The major geology type of the JRW is granite, volcanic tuff, and sandstone [45]. The North River is dominated by granite and sandstone while the West River is mainly comprised of granite and volcanic tuff.

Zhangzhou plain, located at the downstream end of the JRW, constitutes one of China’s most developed regions in terms of agricultural production due to its subtropical monsoon climate and agricultural policies, which are influenced by the closeness to Taiwan [2]. The plain is intensively agricultural with orchards of banana, longan, litchi, pomelo, citrus, and flowers. The N budget for the JRW indicates that fertilizer and animal feedstuff contribute 83.6% of the total N input [46]. For most crop land, surface application of fertilizers is used with high rates in spring (over 200 kg N/hm^2^) and the predominant N fertilizers used in the JRW including urea, ammonium hydrocarbonate and NPK compound fertilizers [45].

The JRW consists mainly of eight counties/districts: Zhangzhou, Xinlou, Zhangping, Hua’an, Changtai, Pinghe, Longhai and Nangjing. More than ten million residents use the Jiulong River as their source of water for residential, industrial and agricultural activities. The watershed’s gross domestic product (GDP) accounts for a quarter of Fujian Province’s economic output while it is approximately one tenth of Fujian Province. Population and economic growth while relatively low municipal wastewater treatment rate make the point source pollution from industrial wastewater and sewage discharged into the river be an innegligible pollution source in the JRW [47]. Additionally, Longyan municipality, including Xinluo and Zhangping, located at the upstream JRW, has the major mining areas of Fujian province. More than 64 kinds of mineral resources can be found there.

### 2.3. Sampling and Experimental Design

Based on the typical land use patterns in the JRW, 20 headwater sub-watersheds classified into three types of groups, namely, natural, urban and agricultural were chosen for sampling. Urban watersheds (U1–U3), agricultural watersheds (A1–A12) and natural watersheds (N1–N5) are the sub-watersheds where the proportion of developed land, cropland and forest land is over 6%, 10% and 80%, respectively. The surface water was sampled during baseflow (i.e. 7-day minimum streamflow) period with nine sampling campaigns from 20 headwater sub-watersheds in three sampling seasons during three consecutive years (Fig. 1). Three investigations were carried out in each of the flood seasons (on August 28^th^, 2010, August 15^th^, 2011, and August 16^th^, 2012), dry seasons (on November 28^th^, 2010, November 29^th^, 2011, and November 20^th^), and transition seasons (on February 28^th^, 2010, March 1^st^, 2011, and March 25^th^, 2012). Note that the 25th and 75th percentile values of the monthly streamflow are usually calculated to define these three seasons, namely, dry season (P<25%), transition season (P = 25%–75%) and flood season (P>75%).

Water quality was characterized using the mean values of these nine-time sampling data sets. To minimum the influence of the sediment and to reduce the influence of hydrologic regime on organic and polyphosphate molecules dissolved in the sample, we sampled the surface water of stream and filtered the water immediately. The samples were kept at 4°C and transported to the laboratory for advanced analysis. Eight chemical parameters were analyzed following standard methods [48] and completed within 24 h after sampling. These parameters were ammonium N (NH_4_^+^-N), the potassium permanganate index (COD_Mn_), soluble reactive phosphate (SRP), nitrate N (NO_3_^–^N), chloride (Cl^−^), sodium (Na^+^), magnesium (Mg^2+^ ) and potassium (K^+^). These parameters were chosen because they can reflect the influence on water quality in terms of natural control (including bedrock geology) and anthropogenic controls (including domestic wastewater/industrial effluents, agricultural activities).

### 2.4. Data Sources

Landscape patterns play an important role in water quality variation at the watershed scale. Land use/land cover (LULC) and landscape pattern metrics (LPMs) were used in this study to delineate the spatial patterns. The spatial pattern of land use and landscape can reflect the underlying human activities [49]. Landsat Thematic Mapper satellite imagery of 2010 with 25 m resolution was used to create LULC data. The land categories were generated using a combination of unsupervised classification and spatial reclassification based on manual on-screen digitizing (for details, see [50]). Land cover was aggregated for six major categories: forest (natural forest, without fertilizing), cropland (including economic forests, with fertilizing), developed land, orchard, water and bare land, and the four most predominant categories (forest, cropland, developed land, and orchard) were retained for analysis in this study. Three LPMs: Patch Density (PD), Largest Patch Index (LPI), and Shannon’s Diversity Index (SHDI), were chosen in this study, to explore the linkage between landscape pattern and water quality.

The natural factors considered in this study were topography and geology. Standard deviation of slope was derived from 25 m-resolution Digital Elevation Model in the JRW. A geologic map in the JRW was generated based on the geology map in Fujian province (scale 1∶1600000), thus two main types of bedrock geology were extracted, namely, sandstones and siltstones (Geology 1), and granites, lavas, and volcanic tuff (Geology 2). Anthropogenic factors also includes socioeconomic development indices represented by population density (Pop_density), GDP, primary industry output value (GDP1), secondary industry output value (GDP2), and tertiary industry output value (GDP3) were collected from the Statistical Yearbook.

### 2.5. Conventional Statistical Analysis

The K independent samples nonparametric test was used to determine the significance of variations of water quality during different sampling seasons. Kruskal-Waillis test was used to calculate mean rank values of each water quality indicator with the following equation:
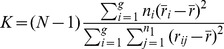
(1)


Where *n_i_* is the number of observations in group; *r_ij_* is the rank of observation *j* from group i; N is the total number of observations across all groups.

The Post Hoc multiple comparisons were used to determine the significance of variations in water quality among headwater watersheds with different dominant land use types. Least Square Difference (LSD) method was used in this study to identify significance of variation in difference sample seasons. The test statistic is calculated as follows:
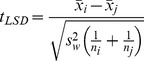
(2)where 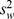 represents the “variance within groups” and is equal to the mean square within in the ANOVA table. This test statistic has N-k degrees of freedom.

Pearson analysis was used in this study to examine the strength and significance of the relationships between selected influencing factors and water quality parameters. Based on a sample of paired data (*x_i_, y_i_*), the sample Pearson correlation coefficient *r* is defined as the following formula:
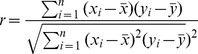
(3)


PCA was used to identify important components that explained most of the variance of water quality in different sampling seasons. This is designed to reduce the number of variables to a small number of indices while attempting to preserve the relationships present in the original data [3]. Kaiser-Meyer-Olkin (KMO) and Bartlett’s test was often used to examine the sensitivity of the data for PCA [9] [51], KMO is a measure of sampling adequacy and a high KMO value (close to 1) generally indicates that PCA may be useful.
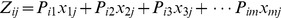
(4)where *Z* is the *PC* value, *P* is the *PC* loading, *x* is the measured value of variable, *i* is the PC number, *j* the sample number, and *m* is the total number of measured variables.

### 2.7. Spatial Statistics

Moran’s I, a global measure of spatial autocorrelation, was used to identify the degree of spatial dependence on water quality parameters over time. Moran’s I is defined as the following formula:
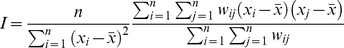
(5)where, *x_i_* and x*_j_* refer to water quality in station *i* and station *j*, respectively 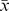 is the overall mean water quality, and w*_ij_* is the weight matrix. Because not all-sub watersheds are adjacent to each other, four nearest neighbors were chosen as cutoff points when creating the weight matrix: that is, if station *i* and *j* are within the neighbor threshold, w*_ij_* = 1, otherwise w*_ij_* = 0 [42]. Global Moran’s I evaluates whether the pattern expressed is clustered, dispersed, or random. A Moran’s I value near +1.0 indicates clustering while a value near –1.0 indicates dispersion, and a value of 0 indicates perfect spatial randomness [52].

To identify landscape factors explaining water quality variations, we used OLS stepwise multiple linear regressions in SPSS. OLS is a type of global statistic, which assumes the relationship under study in constant over space, and so the parameters are estimated to be the same for all the study area. The model can be stated as follows:
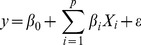
(6)where *y* is the dependent variable, *β_0_* is the intercept, *β_i_* is the parameter estimate (coefficient) for independent variable *x_i_*, *p* is the number of independent variables, and *ε* is the error term.

Compared to the OLS regression models, spatial regression models incorporate spatial dependence in the form of lag or error dependence. The spatial error regression (Eq.7) and spatial lag regression (Eq. 8) are defined as follows:
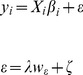
(7)

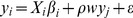
(8)where *y_i_* and *y_j_* represents the dependent variable at sampling site *i* and *j*, *x_i_* is the independent variable at *i*, *β_i_* is the regression coefficient; *ε* is the random error term, *λ* is the spatial autoregressive coefficient of spatial regression, *w_ε_* is the spatially lagged error term, ζ is the homoskedastic and independent error term. ρ is the spatial autoregressive coefficient, and *wy_j_* is the spatially lagged dependent variable.

To identify the influencing factors from the perspective of spatial dependence on river water quality, we compared the OLS models (stepwise multiple linear regression) and spatial regression models by comparison of R^2^, AIC and Moran’s I values in order to select the suitable models. Higher R^2^ in this study meant that influencing factors could explain more variance in the water quality. A lower AIC value suggested that a closer approximation of model to reality and had better model performance. Moran’s I values were used to calculate the residuals from each regression in order to determine whether spatial autocorrelations existed. The dependent variables are mean values of each water quality indicator for all sampling times. Independent variables included the factors derived from the PCA. The same variables were used in both the OLS and spatial regression.

The landscape metrics were calculated using Fragstat 3.3. The calculation of Moran’s I values and GIS analyses were performed using ArcGIS 9.3. OLS and spatial regression were performed in the GeoDa 9.5i. Data pre-processing (including Kaiser-Meyer-Olkin (KMO) and Bartlett’s test) and PCA were conducted using SPSS16.0. Box-plots were produced using Statistical 7.

## Results

### 3.1. Temporal Variation Analyses

#### 3.1.1. Seasonal variation of stream water quality

**Figure 2:**
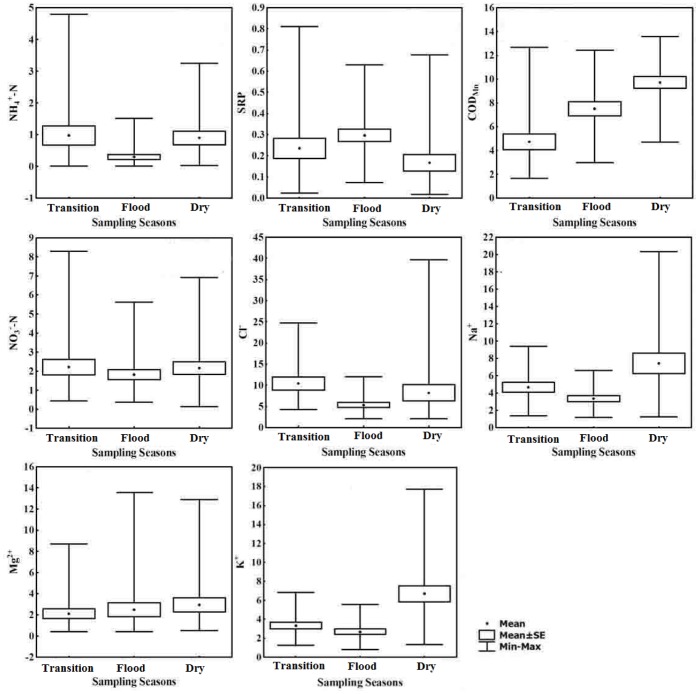
Comparison between concentrations of water quality parameters among the three sampling seasons.

The concentrations of water quality parameters among the three sampling seasons are shown in Fig. 2. All water quality parameters except SRP had their highest values of mean concentration in the dry and transition seasons, whereas the concentrations were the lowest in the flood season. The highest mean concentrations of COD_Mn_, Mg^2+^, Na^+^ and K^+^ were found in the dry season. The mean concentrations of NH_4_^+^-N, NO_3_^–^N, Cl^−^ in the transition season were higher than those in the dry and flood seasons. Interestingly, the highest concentration of SRP was detected in the flood season and the mean concentrations of SRP were in the order of flood season >transition season >dry season.

The K independent samples test to examine the significance of seasonal variations of river water quality (Table 1) shows that the mean rank values for most of the water quality indicators except for SRP and Cl^−^ were higher in the dry season than those in the transition and flood seasons, indicating that the water quality in the dry season was remarkably worse. The mean rank value of Cl^−^ was highest in the transition season while the mean rank value of SRP was highest in the flood season, implying that the most seriously polluted season for Cl^−^ and SRP was the transition and flood seasons, respectively.

**Table 1:** K independent samples of water quality among the different sampling seasons.

	NH_4_^+^-N	SRP	COD_Mn_	NO_3_^–^N	Cl^−^	Na^+^	Mg^2+^	K^+^
Mean Rank(Flood)	23.05	40.40	31.50	28.40	24.20	22.20	28.20	20.70
Mean Rank(Transition)	32.73	29.23	16.95	30.85	38.95	30.45	28.10	26.55
Mean Rank(Dry)	35.73	21.88	43.05	32.25	28.35	38.85	35.20	44.25
Chi-Square	5.755	11.413	22.433	0.498	7.588	9.090	2.173	19.718
Asymp. Sig.	0.056	0.003	0.000	0.780	0.023	0.011	0.337	0.000

Sample No.  = 60; Asymp. Sig. <0.05 indicates significant variation.

#### 3.1.2. Identifying potential pollution sources among three sampling seasons

**Figure 3:**
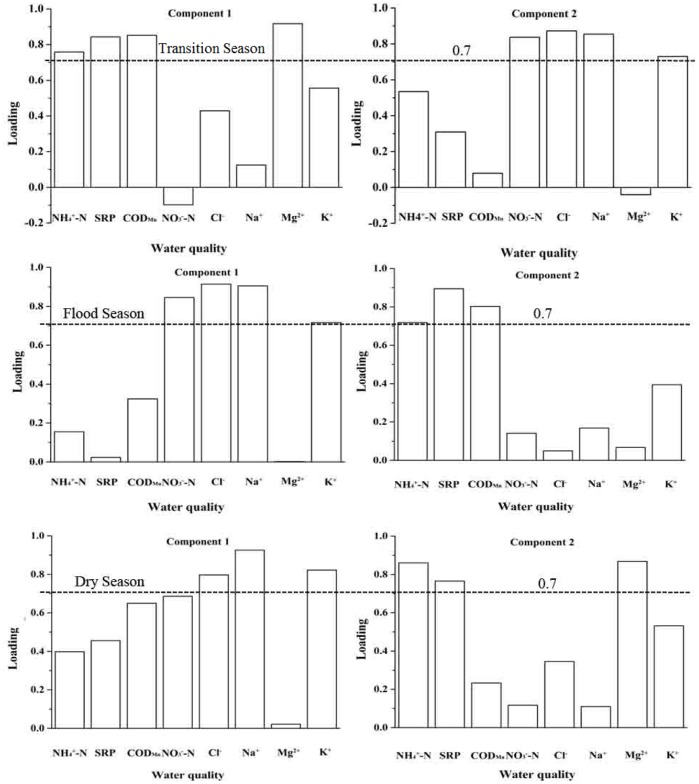
Rotated Component Matrix for water quality parameters among different sampling seasons.

KMO and Barlett’s test was performed before PCA analysis in this study. The KMO value is 0.699 and the p value of Barlet’s test is less than 0.0001, which means there are no significant relationships among water quality variables and it is suitable for PCA analysis. The PCA examining the differences of water pollution characteristics among the three sampling seasons identified the principal components (PCs) with initial Eignen-values >1 (Table 1) and the important water quality parameters with absolute values of component loading >0.7 (Fig. 3). The two PCs identified for the three sampling seasons exhibited distinct differences in terms of the water quality parameters involved (Table 2 and Fig. 3).

**Table 2:** Total variance explained in the three sampling seasons.

Sampling seasons	Component	Initial Eigen-values			Extraction Sums of Squared Loadings			Rotation Sums of Squared Loadings		
		Total	% of Variance	Cumulative%	Total	% of Variance	Cumulative%	Total	% of Variance	Cumulative%
Transition season	1	4.753	59.411	59.411	4.753	59.411	59.411	3.373	42.158	42.158
	2	1.736	21.696	81.107	1.736	21.696	81.107	3.116	38.948	81.107
Flood season	1	3.894	48.677	48.677	3.894	48.677	48.677	3.010	37.630	37.630
	2	1.735	21.690	70.367	1.735	21.690	70.367	2.170	27.123	64.753
Dry season	1	4.800	60.003	60.003	4.800	60.003	60.003	3.427	42.832	42.832
	2	1.185	14.816	74.818	1.185	14.816	74.818	2.559	31.986	74.818

For the transition season, two PCs could explain the majority of total variation (81.107%). PC1, accounting for 42.158% of the total variance, had strongly positive loadings on the organic-related parameters (COD_Mn_), inorganic nutrient-related water quality parameters (NH_4_^+^-N and SRP) and Mg^2+^. Thus, this group might be interpreted as the latent factors from anthropogenic input related to industrial effluents and domestic wastewater, and the natural weathering process. PC2, accounting for 38.948% of the total variance, had strongly positive loadings on NO_3_^–^N, Cl^−^, Na^+^ and K^+^, which might reflect that the impact was associated with agricultural activities.

In the flood season, PC1 explained 37.63% of the total variance and had strongly positive loadings on NO_3_^–^N, Cl^−^, Na^+^ and K^+^, which might have represented the influences of agricultural activities, associated with the precipitation-induced surface runoff. PC2 accounted for 27.132% of the total variance and had strongly positive loadings on NH_4_^+^-N, SRP and COD_Mn_. Thus, PC2 represented industrial effluents and domestic wastewater.

In the dry season, PC1 accounted for 42.832% of the total variance, where Cl^−^, Na^+^ and K^+^ had the greatest loadings. This factor might be interpreted as representing the natural weathering process. PC2 explained 31.986% of the total variance and was largely contributed by nutrients and metal ions related with industrial effluents and domestic wastewater.

### 3.2. Spatial Variation Analyses

**Figure 4:**
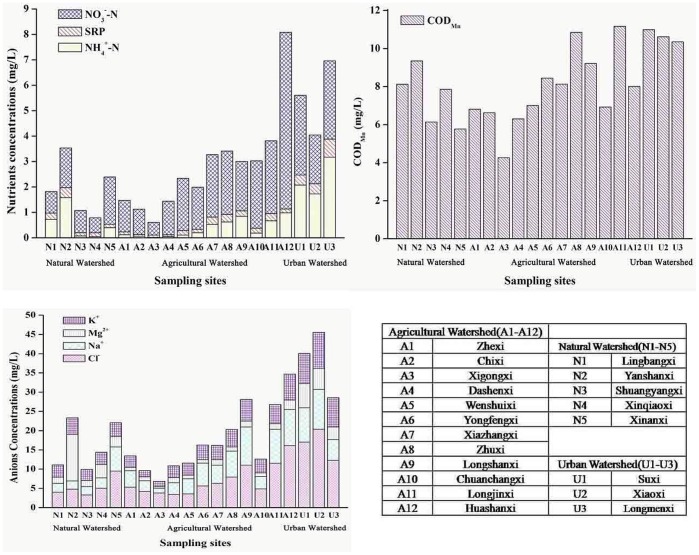
Comparison between concentrations of water quality parameters among the three types of watersheds.

The water quality among agricultural, natural and urban watersheds showed great spatial variations (Fig. 4).

The concentrations of NH_4_^+^-N, SRP, and K^+^ were higher in urban watersheds than those in natural and agricultural watersheds. NO_3_^–^N concentration in agricultural watersheds especially in sub-watershed A12 was generally higher than that in natural and urban watersheds. The concentrations of Cl^−^, and COD_Mn_ were generally higher in urban watersheds than those in natural and agricultural watersheds. It should be noted that the concentrations of Mg^2+^, NH_4_^+^-N, NO_3_^–^N, and COD_Mn_ in sub-watershed N2 were the highest among the natural sub-watersheds, which were even higher than those in some of the sub-watersheds of the urban and agricultural watersheds.

The LSD Post Hoc multiple comparison method used to identify how land use patterns had a clear impact on spatial variations of water quality (Table 3). Table 3 revealed that the urban watersheds exhibited more significance in terms of NH_4_^+^-N, COD_Mn_, Cl^−^, and K^+^ than natural and agricultural watersheds, indicating that urbanized areas were easily exposed to water quality pollution associated with industrial and domestic effluents. The Mg^2+^ concentration in natural watersheds was significantly higher than that in agricultural watersheds, suggesting that the natural weathering process might have played a more important role on Mg^2+^ concentration than agricultural activities. Although most of the water quality parameters collected in agricultural watersheds had higher concentrations than those in natural sub-watersheds, no significant variations were detected.

**Table 3:** LSD Post Hoc multiple comparisons of water quality variables among the three types of watersheds.

Water quality	Urban watersheds-Natural watersheds		Urban watersheds-Agricultural watersheds		Agricultural watersheds-Natural watersheds	
	Mean difference	Sig.	Mean difference	Sig.	Mean difference	Sig.
NH_4_^+^-N	1.757*	0.000	1.958*	0.000	−0.201	0.450
SRP	0.291	0.001	0.329*	0.000	−0.037	0.510
COD_Mn_	3.204*	0.021	2.842*	0.021	0.362	0.699
NO_3_^–^N	1.566	0.136	0.450	0.617	1.116	0.144
Cl^−^	11.245*	0.001	9.593*	0.001	1.652	0.410
Na^+^	5.082*	0.016	2.879	0.104	2.203	0.129
Mg^2+^	0.719	0.668	3.777*	0.019	−3.058*	0.021
K^+^	4.827*	0.000	4.532*	0.000	0.295	0.678

*indicates significant at *p*<0.05.

### 3.3. Spatial and Temporal Variation in Water Quality

Moran’s I values (used to identify the degree of spatial dependence on water quality parameters over time) for the water quality indicators in the three different sampling seasons are shown in Table 4.

**Table 4:** Moran’s I values for water quality indicators among the three sampling seasons.

	NH_4_^+^-N	SRP	COD_Mn_	NO_3_^–^N	Cl^−^	Na^+^	Mg^2+^	K^+^
Flood season	0.81**	−0.21	0.80**	0.03	0.21	0.48*	0.24*	0.66**
Transition season	1.42**	1.39**	0.29	0.18	0.76**	0.33	0.49*	0.99**
Dry season	1.29**	1.28**	0.28	0.31*	0.51**	0.41*	0.47*	1.27**
Mean value	1.35**	1.12**	0.86**	0.19	0.89**	0.47*	0.39	1.32**

**indicates significant at *p*<0.01;

*indicates significant at *p*<0.05.

Significant positive spatial autocorrelations were found for NH_4_^+^-N, Mg^2+^ and K^+^ among the three sampling seasons, indicating that the water quality problem in terms of these three water quality parameters shared regional anthropogenic or natural factors with regards to river pollution over time. The Moran’s I values for most of the water quality indicators (except for COD_Mn_ and Na^+^) in the flood season were the lowest, indicating that significant water quality variations were exhibited between neighboring sub-watersheds and river water quality in the flood season, which might have been contributed by non-point source pollution related to heterogeneous land use patterns in watersheds. The Moran’s I values were highest for NH_4_^+^-N, SRP, COD_Mn_, Cl^−^ and Mg^2+^ in the transition season, and highest for NO_3_^–^N and K^+^ in the dry season, suggesting that most of the water quality parameters in the transition and dry seasons had significant spatial autocorrelations, and exhibited similar levels of water pollution between the neighboring sub-watersheds in these two seasons. Generally, most of the water quality parameters in the dry and transition seasons had more significant spatial autocorrelations than those in the flood season.

### 3.4. Coupled Effects of Natural and Anthropogenic Controls on Water Quality Variations

River water quality is influenced by a combination of natural and anthropogenic factors. In this study, natural factors involved the standard deviation of slope and geology factors, anthropogenic factors included LULC (i.e. four land use types), LPMs (i.e. three landscape indicators), socioeconomic development indices (i.e. Pop_density, GDP, GDP1, GDP2, and GDP3).

#### 3.4.1. General correlation between water quality parameters and environmental variables

Table 5 shows the general correlation between selected influencing factors and water quality parameters using Pearson analysis.

**Table 5:** Correlations between selected water quality parameters and environmental variables using Pearson analysis.

	NH_4_^+^-N	SRP	COD_Mn_	NO_3_^–^N	Cl^−^	Na^+^	Mg^2+^	K^+^
forest	−0.138	−0.117	−0.387*	−0.716**	−0.384*	−0.495*	0.132	−0.314
cropland	−0.254	−0.209	0.024	0.444*	−0.112	0.132	−0.554**	−0.176
developed land	0.769**	0.777**	0.807**	0.441*	0.681**	0.593**	0.376	0.830**
orchard	0.095	0.155	0.383*	0.630**	0.210	0.395*	0.002	0.254
PD	0.140	0.168	0.403*	0.687**	0.328	0.475*	−0.241	0.277
SHDI	0.274	0.242	0.525**	0.741**	0.477*	0.593**	−0.124	0.439*
LPI	−0.139	−0.108	−0.312	−0.651**	−0.328	−0.435*	0.114	−0.294
Pop_density	0.777**	0.701**	0.633**	0.457*	0.879**	0.709**	0.285	0.898**
GDP	0.754**	0.799**	0.706**	0.102	0.406*	0.261	0.552**	0.574**
GDP1	0.246	0.147	0.286	0.782**	0.440*	0.482*	0.036	0.459**
GDP2	0.763**	0.791**	0.637**	0.010	0.374	0.188	0.589**	0.543**
GDP3	0.645**	0.740**	0.760**	0.168	0.377	0.326	0.429*	0.527**
Slope_std	0.501*	0.368	0.381	0.369	0.468*	0.340	0.131	0.472*
Geology 1	0.490**	0.461*	0.116	−0.301	0.108	−0.264	0.684**	0.178
Geology 2	−0.278	−0.144	0.022	−0.243	−0.198	0.045	−0.468*	−0.220

*indicates significant at *p*<0.05;

**indicates significant at *p*<0.01.

- Relationship between water quality and land use/cover: Developed land had positive correlations with most water quality parameters (except for Mg^2+^), suggesting that developed land was an important factor associated with degraded water quality. Forest showed significant negative correlation with COD_Mn_, NO_3_^–^N, Cl^−^ and Na^+^_._ Cropland was significantly positively correlated with NO_3_^–^N, whereas it had significant negative correlation with Mg^2+^. Orchard had significant positive correlation with COD_Mn_, NO_3_^–^N and Na^+^.
- Relationship between water quality and LPMs: PD was significantly positively correlated with COD_Mn_, NO_3_^–^N and Na^+^; And SHDI had significant positive linkages with COD_Mn_, NO_3_^–^N, Cl^−^, Na^+^ and K^+^, whereas LPI had negative associations with most of the water quality parameters.
- Relationship between water quality and social-economic factors: Pop___density had significantly positive correlation with most of the water quality indicators (except for Mg^2+^), similar to build-up. GDP was significant positively related with NH_4_^+^-N, SRP, COD_Mn_, Cl^−^, Mg^2+^ and K^+^_._ Significantly positive relationships between GDP1 and NH_4_^+^-N, SRP, COD_Mn_, Cl^−^, Mg^2+^ and K^+^ were obtained, whereas GDP2 and GDP3 were significantly positively correlated with NH_4_^+^-N, SRP, COD_Mn_, Mg^2+^ and K^+^.
- Relationship between water quality and natural factors: Slope_std had significant negative associations with NH_4_^+^-N, NO_3_^–^N and K^+^, indicating that increased deviation in slope would force the flow to carry pollutants on impermeable surfaces and discharge into river in shorter time. Geology 1 was significantly positively correlated with NH_4_^+^-N, SRP and Mg^2+^. Geology 2 was significantly negatively correlated with Mg^2+^.

#### 3.4.2. Significant explanatory variables identified for variations in water quality

**Figure 5:**
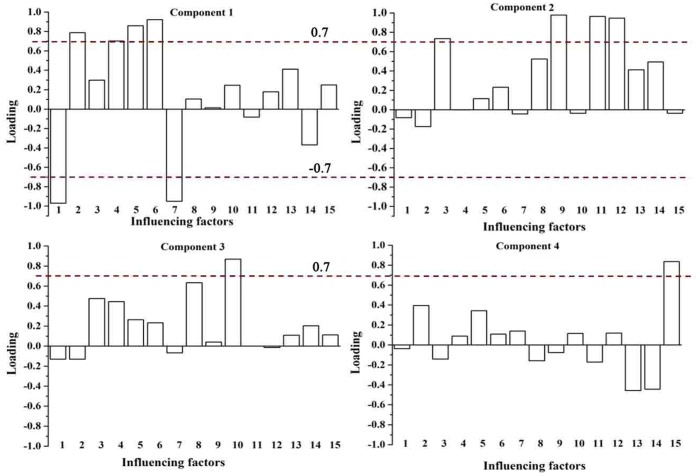
Rotated Component Matrix for environmental variables identified for variation in water quality. (1, 2, 3,4,5,6,7,8,9,10,11,12,13,14,15 stands for forest, cropland, developed land, orchard, PD, SHDI, LPI, Pop_density, GDP, GDP1, GDP2, GDP3, Slope_std, Geology 1, Geology 2).

PCA was used to obtain appreciable data reduction and to identify the important potential factors explaining spatiotemporal variations in water quality. Follow this, OLS and spatial regression were performed to uncover the mechanism of water quality variations from the perspective of spatial dependence of river water quality. The results obtained from PCA are presented in Table 6 and Fig. 5. Table 6 shows the R^2^, AIC and Moran’s I values for water quality residuals for both spatial regression (including spatial lag models and spatial error models) and OLS models.

**Table 6:** Total variance explained for environmental factors.

Component	Initial Eigen-values			Extraction Sums of Squared Loadings			Rotation Sums of Squared Loadings		
	Total	% of Variance	Cumulative %	Total	% of Variance	Cumulative %	Total	% of Variance	Cumulative %
PC1	6.027	40.179	40.179	6.027	40.179	40.179	5.105	34.033	34.033
PC2	4.258	28.385	68.564	4.258	28.385	68.564	4.123	27.489	61.522
PC3	1.226	8.175	76.740	1.226	8.175	76.740	1.807	12.047	73.569
PC4	1.053	7.017	83.757	1.053	7.017	83.757	1.528	10.188	83.757

Four components with initial Eigen-values greater than 1 could explain 83.757% of the total variance (Table 6). PC1, PC2, PC3 and PC4 explained 34.033, 27.489, 12.047 and 10.188% of the total variance. As shown in Fig. 5, PC1 was positively correlated to and largely contributed by cropland, orchard, PD and LPI and negatively affected by forest and LPI. All these important parameters included in PC1 were related to the LULC and LPMs of the watershed, indicating that landscape patterns played an important role in water quality variations in the JRW. PC2 which was positively and largely correlated with developed land, GDP, GDP2 and GDP3, represented the coupled effects of urbanization and socioeconomic development. PC3, which was positively correlated with GDP1, represented the contribution from agricultural activities. PC4 was positively contributed by Geology 2, and this component represented the influence from the natural control. Overall, three potential factors (i.e. PC1, PC2, PC3) identified were related to anthropogenic controls.

Non-significant spatial autocorrelations were found in all models, indicating that both OLS models and spatial regressions could be chosen to determine the relationship between environmental factors and water quality parameters (Table 7). An improvement in R^2^ using the spatial regression model over the OLS model was observed, especially for Cl^−^, Na^+^ and Mg^2+^ (Table 7). In addition, most of the spatial error models had higher R^2^ values than spatial lag models, which suggests that the lower error in the spatial error models. Comparing the AIC values from spatial regressions and those from OLS models, all the spatial error and most of the spatial lag models had lower AIC values than the corresponding AIC values from the OLS models, indicating that spatial regressions, especially spatial error models had better model performance than OLS models.

**Table 7:** Comparison of R^2^, AIC and Moran’s I values between OLS models and spatial regressions.

Water quality parameters		R^2^	AIC	Moran’s I
NH_4_^+^-N	OLS	0.739	31.813	−0.29
	Spatial lag	0.745	33.546	−0.28
	Spatial error	0.793	29.568	−0.24
SRP	OLS	0.716	−34.402	−0.31
	Spatial lag	0.719	−32.54	−0.28
	Spatial error	0.747	−35.500	−0.24
COD_Mn_	OLS	0.711	67.929	−0.25
	Spatial lag	0.713	69.856	−0.25
	Spatial error	0.763	65.436	−0.12
NO_3_^–^N	OLS	0.767	50.399	0.06
	Spatial lag	0.833	47.305	−0.16
	Spatial error	0.846	45.967	0.12
Cl^−^	OLS	0.587	113.39	−0.25
	Spatial lag	0.797	106.346	−0.25
	Spatial error	0.707	110.047	0.05
Na^+^	OLS	0.530	93.660	−0.17
	Spatial lag	0.664	91.319	−0.15
	Spatial error	0.647	90.483	−0.08
Mg^2+^	OLS	0.360	96.857	−0.41
	Spatial lag	0.365	98.766	−0.39
	Spatial error	0.568	92.963	−0.39
K^+^	OLS	0.774	65.727	0.09
	Spatial lag	0.869	60.085	−0.24
	Spatial error	0.833	62.930	0.16

Given the higher R^2^ values and lower AIC values from spatial regression model, spatial regression model were chosen for further study to identify significant explanatory variables for each parameters. The models explain approximately up to 87% of the variation in water quality (Table 8).

**Table 8:** Spatial regression models established in the JRW.

Water quality parameters	Spatial Regression models	R^2^	Sig.
NH_4_^+^-N^a^	y = 0.710+0.650*factor2+0.264*factor3–0.236*factor4 (LAMBDA = −0.561)	0.793	**
SRP^a^	y = 0.233+0.128*factor2+0.033*factor3(LAMBDA = −0.395)	0.747	*
COD_Mn_^a^	y = 8.194+0.654*factor1+1.504*factor2+0.597*factor3 (LAMBDA = −0.452)	0.763	**
NO_3_^–^N^a^	y = 2.043+0.774*factor1+0.828*factor3 (LAMBDA = 0.709)	0.846	**
Cl^−b^	y = 14.456+3.878*factor2+4.058*factor3–0.799*WY	0.797	**
Na^+a^	y = 8.195+1.229*factor1+1.182*factor2+2.155*factor3–0.567*WY	0.664	**
Mg^2+a^	y = 2.511–0.883*factor1+1.561*factor2–0.804*factor4(LAMBDA = −0.716)	0.568	*
K^+b^	y = 7.207+2.032*factor2+1.772*factor3–0.380*factor4–0.656*WY	0.869	*

Note: Factor1, 2, 3, and 4 corresponds to the four components identified and presented in Fig. 6.

*a* denotes the results of spatial error models, *b* denotes the results of spatial lag models.

WY: weighted mean of the dependent variable for adjacent sub-basins.

*indicates significant at *p*<0.05.

**indicates significant at *p*<0.01.

As shown in Table 8, factor2 and factor3 were the two most important factors associated with water quality variations, implying that anthropogenic controls played critical roles in variations of water quality in the JRW. Factor2 was related to all the water quality parameters except for NO_3_^–^N while factor3 was included in all the regression models except for Mg^2+^. In particular, factor2 had significant impact on NH_4_^+^-N, SRP, COD_Mn_, Mg^2+^ and K^+^, implying environmental factors related to urbanization and socioeconomic development were the important explanatory variables for these parameters. It is understandable that NO_3_^–^N was largely and positively contributed by factor3 associated with agricultural activities. Factor1 representing landscape pattern was also an important factor, which had significant positive effects on COD_Mn_, NO_3_^–^N, and Na^+^. Factor4 appeared in three of the regression models related to NH_4_^+^-N, Mg^2+^ and K^+^, and the relationships were significantly negative.

Generally, agricultural activities and landscape patterns had coupled effects on variations related to NO_3_^–^N and Na^+^. Urbanization and socioeconomic development were the dominant explanatory variables for COD_Mn_ and NH_4_^+^-N were also the primary predictor for SRP, Mg^2+^, and K^+^. This suggested that urban development was the driving source of oxygen demand and nutrient concentration. Agricultural activities and urbanization and socioeconomic development were the two important predictors for Cl^−^.

**Figure 6:**
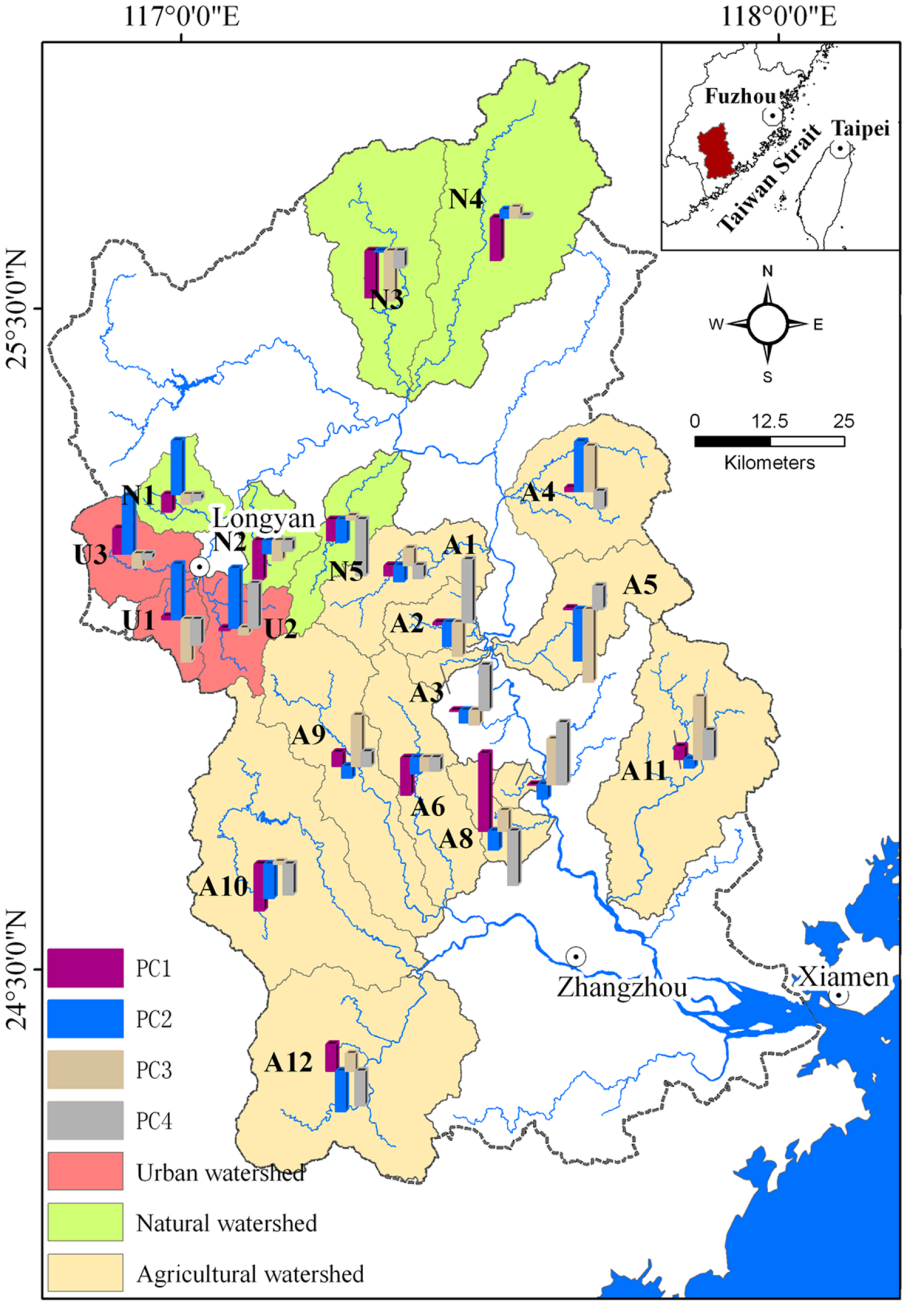
The four potential pollution sources identified to explain spatiotemporal variations in water quality for 20 headwater watersheds in the JRW. (PC1, PC2, PC3, and PC4 represents landscape patterns, urbanization and socioeconomic development, agricultural activity, and natural control, respectively).

Fig. 6 shows the four potential factors identified to explain spatiotemporal variations in water quality for 20 headwater watersheds in the JRW. Compared to other two types of watershed, PC2 (i.e. urbanization and socioeconomic development) is the largest contributor for variations among four factors identified in three urban watersheds. In terms of natural watersheds, environmental factors mostly related to anthropogenic controls had a negative influence on water quality.

## Discussion

### 4.1. Causal Factors of Spatiotemporal Variations in Water Quality

Water quality is affected by the combination of natural and anthropogenic factors, the relative influences of which change with temporal and spatial scale [1]. Our study effectively differentiated the impacts from anthropogenic factors (including landscape pattern, agricultural activities, urban and socioeconomic development) and natural factors on stream water quality. Our results showed that urban and socioeconomic development acted as the primary predictor for COD_Mn_ and NH_4_^+^-N, and the relationships between developed land and water quality parameters were consistently positive. Many previous studies had similar findings concerning the contribution of urbanization to degraded water quality [2], [43], [53]–[54]. Our findings also supported prior observations concerning more pollutants in urbanized areas with higher population density [54]–[55] and the potential occurrence of water quality pollution under rapid economic development [56]–[57]. Moreover, our study also found that the highest concentration of Cl^−^ in urban watershed, which indicated the potential risk of increasing salinity in urban areas [1], [57]–[58].

The percentage of cropland was significantly positively correlated with NO_3_^–^N and agricultural activities were identified in this study as an important predictor for NO_3_^–^N through the spatial regression model, which verified that cropland and agricultural activities were an important “source” for NO_3_^–^N..Kannel et al. (2007) also note that the increasing concentration of NO_3_^–^N with intensified use of chemical fertilizers together with urbanization [59]. In our study, the percentage of orchard was significantly positively correlated with COD_Mn_, NO_3_^–^N, and Na^+^. As one of China’s most developed areas in terms of agricultural production, orchards are intensive especially in the downstream of the JRW, therefore, a large amount of animal waste and organic fertilizer used in the orchards, together with the rotten fruits, would make a contribution to the organic pollutants and NO_3_^–^N. Other studies in the JRW also found that large amount of nitrogen fertilizers are applied to crop which increase the source of nitrogen to stream [45]–[46]. The result from the spatial regression analysis in this study also suggested that agricultural activities were the important predictor for water quality variation especially Na^+^ and Cl^−^ (Table 8). In this study, the percentage of forest had negative correlations with most of the water quality parameters, which was consistent with the previous findings that forest possesses the ecological function of filtering pollutants [29], [60].

In terms of natural factors, significantly positive relationships between Slope_std and NH_4_^+^-N, Cl^−^ and K^+^ were found in this study. Slope represents an aggregation of rainfall, soil, geology, vegetation and temperature [12]. Similar observations were obtained by other researchers that slope variable act as a sink for some water quality parameters [29], [41]–[42]. In this study, significantly positive correlation was found between Geology 1 (sandstones and siltstones) and NH_4_^+^-N, SRP, Mg^2+^ as the result of weathering and dissolution of minerals from the local agricultural soil and bedrock [61]. On the other hand, Geology 2 (granites, lavas, and volcanic tuff) was negatively correlated with Mg^2+^ since the granitic bedrock is too hard to be weathered and the negative effect of pore solution by the volcanic tuff on Mg^2+^ release [62]–[63]. In this study, we found that Mg^2+^ concentration in natural watersheds was significantly higher than that in agricultural watersheds. Pearson analysis also showed that the correlation between concentration of Mg^2+^ and Geology 1 were stronger (r = 0.684, *p*<0.01) than with other factors (Table 5). Rothewell et al. (2010) had a similar observation that the Mg^2+^ concentration is related to underlying geology rather than the effect of human activities [12].

### 4.2. Spatial Dependence of Water Quality in Different Seasons

River water quality exhibits spatial autocorrelation, since adjacent sites are dominated by similar natural ecosystems and experience comparable human disturbances [42]–[43]. Our results showed that global Moran’s I values for most of the water quality parameters (except for COD_Mn_ and Na^+^) were lowest in the flood season, whereas most water quality parameters showed significant spatial autocorrelation in the dry season. In the flood season, nonpoint source pollution constituted the primary pollution source (suggested in subsection 4.1.2) and we inferred hydrological process, particularly agricultural and urban stormwater runoff containing different amounts of nutrients from different land cover types such as cropland and street, might make different contribution to water quality degradation in sub-watersheds with great spatial heterogeneity. Thus, it might result in the water quality problems becoming more localized. In contrast, river water quality in the dry season was mostly affected by point source pollution such as industrial and domestic effluents, suggesting that water quality is a reflection of regional anthropogenic activities or natural factors [42]. In addition, NH_4_^+^-N had the highest Moran’s I value among all the water quality indicators, which was further verified the results from the spatial regression model, which indicated that NH_4_^+^-N was more associated with point source pollution, such as industrial effluents and domestic wastewater. In other words, watersheds with similar urbanization and socioeconomic development might show a similar NH_4_^+^-N pollution pattern. NH_4_^+^-N and COD_Mn_ are two important indicators which can to some extent reflect the local wastewater treatment capacity [2], [64], and improving the capacity for industrial and domestic wastewater treatment is important for regional water management in the context of accelerating urbanization. Comparatively, NO_3_^–^N had the lowest Moran’s I value in our study, indicating that NO_3_^–^N pollution was localized. NO_3_^–^N was largely contributed by agricultural activities and natural factors including soil, geology and slope can also affect the pollutant export through hydrologic pathways such as runoff to a water body [29].

### 4.3. Seasonal Pattern and the Underlying Mechanisms

Most seasonal variations in river water chemistry are driven by climatic and biotic factors and are therefore largely governed by the watershed processes related to natural or human induced disturbances [29], [65]. The water soluble nutrients such as inorganic N may be leaching into the groundwater and transport into stream via subsurface water [66]. However, insoluble nutrient species such as P often as the form of particulates, thus transporting with sediments through the surface water pathways especially in the rainy day [67]–[68]. As a result, bioreactive elements can cycle differently and undergo release or retention from sediments in response to shifting redox conditions and warming. Thus, the source of nutrients can be variable, for example during flood season, high nitrate concentration in streamwater results from higher contribution of nitrate-rich hillslope shallow groundwater, whereas during dry season streamwater mainly comes from denitrified bottomland groundwater [69]–[70] and deep fractured aquifers [71].

In our study, the pronounced seasonal changes in NH_4_^+^-N, NO_3_^–^N, Cl^−^, COD_Mn_, Mg^2+^, Na^+^ and K^+^ concentrations in the 20 headwater streams (Fig. 2 and Fig. 4), with highest concentrations in the dry and transition seasons and lowest concentrations in the flood season, suggests that river water quality dynamics in the JRW may be determined by the anthropogenic controls such as domestic wastewater & industrial effluent, and agricultural N input associated with vadose zone leaching and groundwater seepage, as well as lowest in-stream N immobilization rate due to lowest temperature in the dry and transition seasons. Besides, increased streamflow in the flood season may reduce the concentration if the source of nutrients is invariant, thus effective dilution effect associated hydrologic regime might lead to the lowest concentrations in the flood season in the JRW. Potential pollution sources identified by PCA analysis in subsection 4.1.2 could also interpret for the seasonal variations in river water quality. Point source pollution associated with industrial and domestic wastewater was identified as the important potential factor to make a great contribution to the variations in NH_4_^+^-N and COD_Mn_, and agricultural activities were the primary predictor for NO_3_^–^N (Table 8).

In this study, SRP concentration is highest in the flood season (in Aug.), which might be related to more sediment-bound P input associated with hydrological processes such as stormwater runoff in this season. The SRP load exported from catchment non-point sources was associated with stormwater runoff and released from in-stream internal sources with the increasing river flow [2], [72]. As the result of that, the net effect of the amount of P entering and leaving an ecosystem as quantified by a mass balance provides a measure of P retention (e.g. during low stream flow) or P export (e.g. during high stream flow) over a given spatial and temporal scale [73]. It should be noted that other studies found SRP can be controlled by the biogeochemical process and anthropogenic input. Duan et al. (2012) notes the effect of temperature on seasonal change SRP based on the observation that high SRP concentrations exhibit in the low flow during summer as result of biochemical reactive [13]. Bowes et al. (2005) found that streams receiving wastewater effluent typically show a characteristic pattern of high P concentration during summer low flow and more diluted concentration during winter storm events in an English catchment [34]. Hydrological process rather than biogeochemical process drives the seasonal variations of P export in the JRW, which might be due to greater variability of inter-annual precipitation than of inter-annual temperature in the watershed studied.

### 4.4. Spatial Variations of Water Quality among three Types of Watershed

Land use and land cover play a central role in fate and transport of water quality [48], [74]. Runoff from agricultural land and effluents from urban and industrial area are major sources of nutrients and fine sediment in river systems [26], [75]–[76]. Urbanization and agricultural activities are common source of elevated water pollution, which is expected to continue increasing in the coming decades due to growing populations, further development and greater demand for food production [1], [13], [77]. Our study showed that the water in urban watersheds was more seriously polluted than in agricultural and natural watersheds, and the natural watersheds often had better water quality. This was not surprising due to the fact that the selected urban sub-watersheds had the highest urbanization level and there are many mining lots and steel plants located at the upstream of these watersheds. Acid mine drainage from active and abandoned strongly influences stream water chemistry by acidifying stream water which in turn increase the dissolution of mineral and high concentrations of several elements [78]. In addition, livestock farming was also intensive there. As a result, the water quality in the urban watersheds was influenced by a combination of point source and non-point source pollution associated with industrial, domestic and livestock waste. Additionally, most of the water quality parameters in agricultural watersheds had higher concentrations than those in natural watersheds, but no significant variance was obtained. This could be explained by the fact that the amount of natural watersheds is smaller than that of agricultural watersheds and natural sub-watershed, N2 was a special sub-watershed that had a high percentage of forest, but was seriously polluted by mining activities.

### 4.5. Implications for Water Quality Management

The findings of our study are helpful for water resource management within the watershed. Water quality was worst in the dry season and point source pollution associated with industrial and domestic wastewater made a great contribution to the variations of NH_4_^+^-N and COD_Mn_ in this season. Cl^−^ was relatively higher in urban watersheds than those in natural and agricultural watersheds, indicating the potential increasing salinization in urbanized areas. Moreover, the most significant spatial autocorrelation for most water quality parameters found in the transition and dry seasons, especially in the dry season. Thus, regional water management alternatives such as improving the capacity of wastewater treatment are more suitable than localized measures in the dry season. Comparatively, nonpoint source pollution associated with agricultural activities made the greatest contribution to the spatiotemporal variations in water quality in the flood and transition seasons. Water quality problems become more localized in the flood season due to the fact that agricultural nonpoint source pollution in different watersheds might make a different contribution to water quality degradation in sub-watersheds with great spatial heterogeneity. Thus, more attention should be paid to agricultural non-point source pollution and more localized water management measures should be taken in the flood season. Considering that urbanization and socioeconomic development and landscape pattern were the two dominant influencing factors for water quality variation, spatial planning for urbanization and socioeconomic development at watershed scale should be recognized by the local government when balancing economic growth and environmental conservation.

## Conclusion

Anthropogenic input related to industrial effluents/domestic wastewater, agricultural activities associated with the precipitation-induced surface runoff, and natural weathering process were identified as the potential important factors to drive the seasonal variations in stream water quality in 20 headwater watershed of the JRW for the transition, flood and dry seasons, respectively. The NH_4_^+^-N, NO_3_^–^N, Cl^−^, COD_Mn_, Mg^2+^, Na^+^ and K^+^ in the 20 headwater streams had the highest concentration concentrations in the dry and transition seasons and lowest concentrations in the flood season. SRP had the highest concentrations in the flood season as a result of hydrological control.

Anthropogenic activities and watershed characteristic led to the spatial variations in stream water quality in three types of watersheds. Anthropogenic input associated with industrial effluents/domestic wastewater led to the concentrations of NH_4_^+^-N, SRP, K^+^, COD_Mn_, and Cl^−^ were generally higher in urban watersheds than those in natural and agricultural watersheds. NO_3_^–^N were generally higher in agricultural watersheds than those in urban and natural watersheds as a result of anthropogenic input related to agricultural activities. Mg^2+^ concentration in natural watersheds was significantly higher than that in agricultural watersheds, which is largely due to the watershed characteristic (bedrock geology). Spatial autocorrelations analysis showed similar levels of water pollution between the neighboring sub-watersheds exhibited in the dry and transition seasons while non-point source pollution contributed to the significant water quality variations between neighboring sub-watersheds.

Spatial regression analysis showed anthropogenic controls played critical roles in variations in stream water quality in the JRW. Urbanization and socioeconomic development were the dominant explanatory variables for NH_4_^+^-N, SRP, COD_Mn_, Mg^2+^, Cl^−^ and K^+^, and agricultural activities and landscape patterns had coupled effects on variations related to NO_3_^–^N and Na^+^. Management implications were further discussed for water resource management. This research demonstrates that the coupled effects of natural and anthropogenic controls, together with underlying hydrologic and biogeochemical processes, contribute to the seasonal and spatial variation of headwater stream water quality in a coastal watershed with high spatial variability and intensive anthropogenic activities.
